# Enzymatic Modification of *Porphyra dioica*-Derived Proteins to Improve their Antioxidant Potential

**DOI:** 10.3390/molecules25122838

**Published:** 2020-06-19

**Authors:** Filipa B. Pimentel, Maria Cermeño, Thanyaporn Kleekayai, Pádraigín A. Harnedy-Rothwell, Eduarda Fernandes, Rita C. Alves, M. Beatriz P.P. Oliveira, Richard J. FitzGerald

**Affiliations:** 1 REQUIMTE/LAQV, Department of Chemical Sciences, Faculty of Pharmacy, University of Porto, 4050-313 Porto, Portugal; beatoliv@ff.up.pt; 2 Proteins and Peptides Research Group, Department of Biological Sciences, University of Limerick, V94 T9PX Limerick, Ireland; maria.cermeno@ul.ie (M.C.); thanyaporn.kleekayai@ul.ie (T.K.); padraigin.harnedy@ul.ie (P.A.H.-R.); dick.fitzgerald@ul.ie (R.J.F.); 3 REQUIMTE/LAQV, Laboratory of Applied Chemistry, Department of Chemical Sciences, Faculty of Pharmacy, University of Porto, 4050-313 Porto, Portugal; egracas@ff.up.pt

**Keywords:** *Porphyra dioica*, antioxidant activity, enzyme-assisted hydrolysis, reactive oxygen species, seaweed

## Abstract

Enzymatic hydrolysis has been employed to modify protein functional properties and discover new sources of antioxidants. In this study, the effect of different enzymatic treatments on antioxidant activity of *Porphyra dioica* (blades and protein isolate (PI)) was investigated. Protein nitrogen content of *P. dioica* blades and PI were 23 and 50% (dry weight), respectively. Blades and PI were hydrolyzed with Prolyve® and Prolyve® plus Flavourzyme®. Peptide profiles and molecular mass distribution of the hydrolysates were investigated. The hydrolysis promoted generation of peptides and low molecular mass components <1 kDa. Antioxidant activity was assessed using ferric reducing antioxidant power (FRAP), 2,2-diphenyl-1-picrylhydrazyl (DPPH·) scavenging, 2,2′-azinobis-(3-ethylbenzothiazoline-6-sulfonate) (ABTS·+) inhibition, and reactive oxygen species scavenging ability, i.e., oxygen radical absorbance capacity (ORAC) and hypochlorous acid (HOCl) scavenging assays. In general, enzymatic hydrolysis of *P. dioica* blades and PI enhanced the in vitro antioxidant activity. Direct hydrolysis of blades improved ORAC values up to 5-fold (from 610 to 3054 μmol Trolox eq./g freeze dried sample (FDS). The simultaneous release of phenolic compounds suggested a potential synergistic activity (ORAC and ABTS·+ assays). Such hydrolysates may be of value as functional food ingredients.

## 1. Introduction

In living systems, free radicals and other reactive oxygen species (ROS) are products of normal cellular metabolism. These molecules can play a dual role. At low/moderate concentrations, they can act as molecular signals that activate beneficial stress responses. However, at high levels, potential oxidative damage and tissue dysfunction may occur [1]. From another perspective, oxidation is also a major cause of foodstuff quality loss [2]. The oxidation process can generate low-molecular-weight off-flavor compounds which may reduce consumer acceptance. Furthermore, essential nutrients may be degraded and toxic compounds (e.g., dimers or polymers of lipids and proteins) may be produced following extensive oxidation [3,4]. Antioxidants can delay oxidative reactions by several mechanisms. These may include a combination of free radical scavenging, singlet oxygen quenching, pro-oxidative metals chelation and/or lipoxygenase inactivation [3]. There has been an increased interest in identifying naturally-occurring compounds with antioxidant properties as alternatives to synthetic products [5], since they can be highly valued by the food, nutraceutical and/or pharmaceutical industries.

Seaweed proteins have been investigated in recent years due to their potential antioxidant properties [6]. Additionally, a diversity of biological activities has been attributed to seaweed protein hydrolysates which highlights their potential as functional ingredients [4,7]. For instance, hydrolysates have the ability to inhibit oxidative reactions by interacting or neutralizing free radicals, leading to the development of novel food ingredients of special interest for health promotion and disease prevention [8]. Enzyme-assisted protein hydrolysis using exogenous proteases is a widely used process to generate food-derived hydrolysates [9] which present many advantages over other processes such as chemical hydrolysis. For instance, it requires milder processing conditions, e.g., pH and temperature, allowing for better control of the reaction and the acquisition of well-defined peptide profiles without the formation of undesirable substances [10]. Furthermore, depending on the enzymes used, the amino acid composition equivalent to the starting protein substrate is maintained [9,11]. Moreover, it is safer as it does not involve organic solvents or toxic chemicals, making it suitable for food/nutraceutical applications [9]. However, this process often involves a pre-treatment, i.e., protein isolation, in order to remove other compounds such as carbohydrates or lipids [12].

*Porphyra* sp., commonly known as nori, is one of the most valuable red seaweed species, and it is well recognized for its high protein content (~45% dw) [13,14]. Hydrolysates from *Porphyra sp.* protein isolates have been shown to exert antioxidant activity [15,16,17,18,19,20,21,22]. Within *Porphyra* sp., *Porphyra dioica* is widely distributed and cultivated. A limited number of studies using *P. dioica* as a source of bioactive peptides have been reported to date [23]. Stack, et al. [24] reported higher antioxidant activity for hydrolysates obtained from *P. dioica* protein isolate compared to other red seaweed species such as *Palmaria palmata* [25,26]. Furthermore, Pimentel, et al. [27] reported that simulated gastrointestinal digestion of both *P. dioica* derived biomass and protein isolate improved antioxidant activity of the resultant hydrolysates. However, only a few studies have investigated direct enzymatic hydrolysis of the *P. dioica* biomass (blades).

Therefore, the aim of this study was to assess the effect of enzymatic hydrolysis (using a single or two sequential food-grade proteolytic preparations) on the antioxidant activity of *P. dioica* blades and its protein isolate.

## 2. Results and Discussion

Two substrates were used in this study: The complete biomass (blades) and the protein isolate obtained therefrom (PI; obtained following alkaline extraction and isoelectric precipitation at pH 4.5). Proteins were extracted and precipitated prior to enzymatic hydrolysis in order to remove other compounds, i.e., polysaccharides or polyphenols, that may interfere with algal protein digestion [28].

### 2.1. Protein Content

Table 1 shows the nitrogenous fractions and protein content of the blades and the PI samples. As expected, the total nitrogen (TN) and protein nitrogen (PN) content was ~2-fold higher in the PI than in the blades. The protein content was estimated to be 23.09 ± 0.90 and 50.30 ± 1.52 for the blades and PI, respectively using a nitrogen to protein conversion factor of 5.00 (Table 1), as described in previous studies for seaweed [29].

### 2.2. Physicochemical Characterization of Protein Hydrolysates

Protein hydrolysates of the *P. dioica* PI were generated using Prolyve® 1000 and Flavourzyme®. Prolyve® 1000 is a non-specific microbial endoproteinase with subtilisin activity, produced by *Bacillus licheniformis*. Flavourzyme® is a fungal enzyme complex produced by *Aspergillus oryzae* that possesses mainly exopeptidase activity. Prolyve® 1000 was utilized in the first instance to hydrolyze proteins to low molecular mass peptides. The subsequent inclusion of Flavourzyme® for additional 120 min aimed to promote further peptidase mediated breakdown of peptides released by Prolyve® 1000. Similar combination of enzymes has been used previously with other substrates to reduce bitterness due to the action of exopeptidase activity [30,31]. However, to the best of our knowledge, the combination of Prolyve® 1000 and Flavourzyme® has not been previously used in the generation of hydrolysates from *P. dioica*. For both substrate samples, controls were included which involved incubation of the substrate under the same conditions (pH, temperature, and time), except that no proteases were added to the reaction.

**Figure 1:**
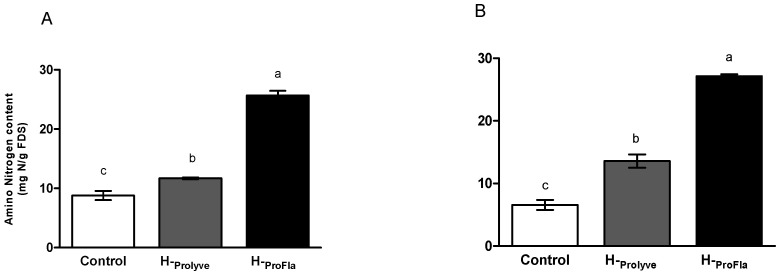
Amino nitrogen content liberated from *Porphyra dioica* blades (**A**) and protein isolate (**B**), following 2 h hydrolysis at 50 °C with Prolyve® 1000 (H-Prolyve), and 4 h with Prolyve® 1000 plus Flavourzyme® (H-ProFla). Values represent mean ± SD (*n* = 3). Results are expressed as mg of amino nitrogen per g of freeze-dried sample (mg N/g freeze dried sample (FDS)). Different letters (a, b and c) denote significant differences at *p* < 0.05 for each sample.

Trinitrobenzenesulphonic acid (TNBS) reacts with primary amino groups. Analysis with TNBS allows to estimate the extent of hydrolysis by quantification of free N-terminal amino groups released during hydrolysis [32]. As shown in Figure 1, hydrolysis promoted the release of amino nitrogen in the blade (A) and in PI (B) samples, when comparing to their respective controls and corresponding hydrolysates. An increase in the concentration of free amino nitrogen was observed following hydrolysis with Prolyve® 1000 (H-Prolyve), which increased considerably following incubation with Flavourzyme® (H-ProFla): 8.79 ± 0.77 vs. 11.69 ± 1.45 vs. 25.69 ± 0.77 and 6.57 ± 0.81 vs. 13.56 ± 1.08 vs. 27.14 ± 0.31 mg N/g sample, for control vs. H-Prolyve vs. H-ProFla, for blade and hydrolysates, respectively). These values were higher than those obtained in a previous study in which Alcalase® (*Bacillus licheniformis* protease) combined with Flavourzyme® were used to generate hydrolysates from *P. dioica* protein isolates (23.95 ± 0.42 mg N/g sample). The reported values of free amino nitrogen released following hydrolysis ranged from 2.57 to 23.95 mg amino nitrogen/g of freeze-dried powder [24].

**Figure 2:**
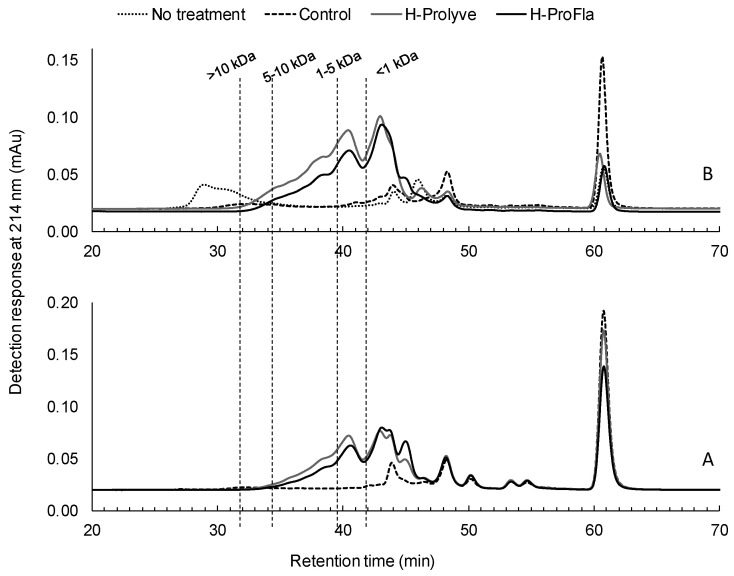
Gel permeation high-performance liquid chromatography profiles of *Porphyra dioica* blades (**A**) and protein isolate (**B**) following 2 h hydrolysis at 50 °C with Prolyve® 1000 (H-Prolyve) and a 4 h hydrolysis with Prolyve® 1000 plus Flavourzyme® (H-ProFla). No treatment: no enzyme addition without incubation at 50 °C for 4 h; Control: samples incubated at 50 °C for 4 h without enzymes. Dashed vertical lines indicate the retention times corresponding to proteins and peptides with masses <1 kDa, 1–5 kDa, 5–10 kDa, and >10 kDa.

The molecular mass distribution of the soluble proteinaceous components in the unhydrolyzed samples (controls) and the respective hydrolysates was analyzed by gel permeation high performance liquid chromatography (GP-HPLC). Distinct peptide profiles were observed in the blade and the PI samples as depicted in Figure 2 A,B, respectively. Changes in the GP-HPLC profiles of the hydrolysates compared to the corresponding controls demonstrated that enzymatic treatment promoted hydrolysis of the samples with a larger proportion of lower molecular mass peptides (<1 kDa) in H-Prolyve and H-ProFla. This is desirable, as it has been reported that low molecular mass peptides can be absorbed easily in the gastrointestinal tract [33].

**Figure 3:**
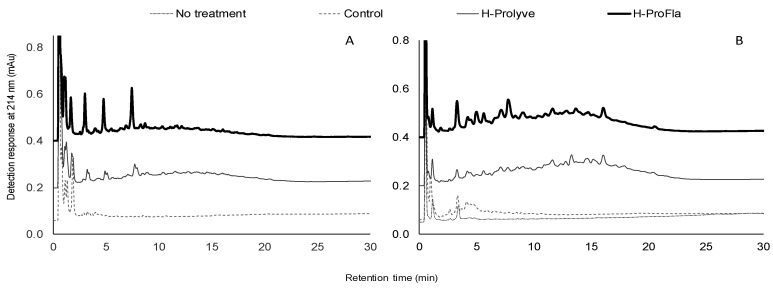
Analytical reverse-phase ultra-performance liquid chromatography profiles of blades (**A**) and protein isolate (**B**) of *Porphyra dioica* following 2 h hydrolysis at 50 °C with Prolyve® 1000 (H-Prolyve) and a 4 h hydrolysis with Prolyve® 1000 plus Flavourzyme® (H-ProFla). No treatment: no enzyme addition without incubation at 50 °C for 4 h; Control: samples incubated at 50 °C for 4 h without enzymes.

Major changes can also be observed in the RP-UPLC profiles of the hydrolysates of both samples (blades and PI) following incubation with both proteinases (Figure 3). Differences in the specificity of the proteinases for substrates resulted in distinct peptide profiles. The generation of small molecular mass and more hydrophilic peptides can be observed in the H-Prolyve and H-ProFla profiles of both samples (Figure 3). Interestingly, in the blade sample (Figure 3A), the peaks with higher intensity eluted mainly in the first 10 min. In the PI hydrolysates (Figure 3B), the intensity of the peaks was lower, but a larger number of peaks appeared in the chromatograms, which in turn, were eluted over a longer period of time (up to 20 min).

### 2.3. Antioxidant Activity

The antioxidant activity of the samples was determined using a range of in vitro antioxidant assays with different reaction mechanisms including ferric reducing capacity (FRAP) and radical scavenging assays, i.e., 2,2-diphenyl-1-picrylhydrazyl (DPPH·) scavenging, 2,2′-azinobis-(3-ethylbenzothiazoline-6-sulfonate) (ABTS·+) inhibition, oxygen radical absorbance capacity (ORAC) and hypochlorous acid (HOCl) inhibition assay. Although DPPH· and ABTS·+ are widely used to assess antioxidant activity of food proteins, they are not considered physiologically relevant to biological systems [34]. Therefore, the scavenging capacity against biological relevant ROS such as peroxyl radical (ROO·) and HOCl were also evaluated (by the ORAC and HOCl inhibition assay, respectively).

**Figure 4:**
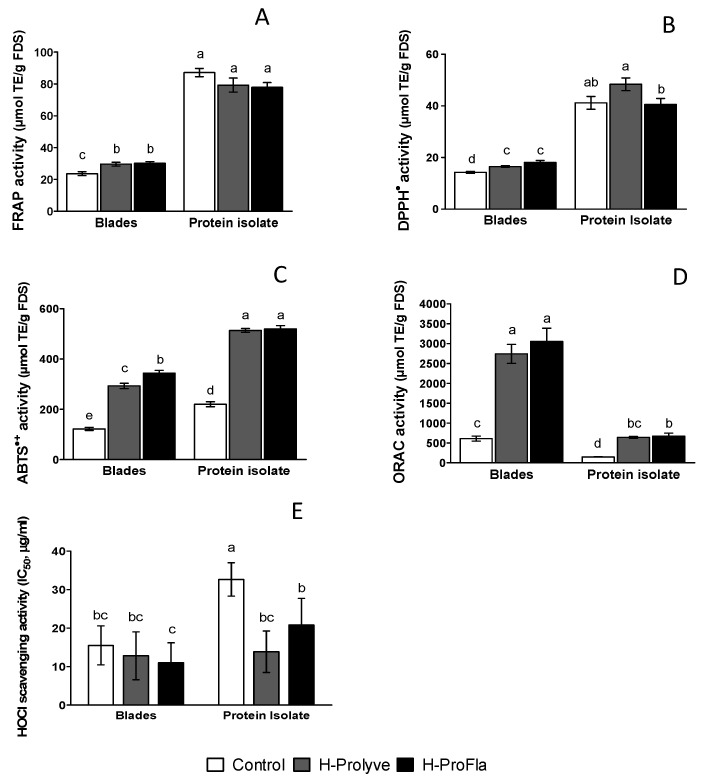
Ferric reducing antioxidant power (FRAP; **A**), 2,2-diphenyl-1-picrylhydrazyl inhibition (DPPH·; **B**), 2,2′-azinobis-(3-ethylbenzothiazoline-6-sulfonate) inhibition (ABTS·+; **C**), oxygen radical absorbance capacity (ORAC; **D**) and hypochlorous acid (HOCl; **E**) scavenging activity of *Porphyra dioica* blades and protein isolate, following 2 h hydrolysis at 50 °C with Prolyve® 1000 (H-Prolyve), and 4 h with Prolyve® 1000 plus Flavourzyme® (H-ProFla). For FRAP, DPPH·, ABTS·+ and ORAC assays, results (mean ± standard deviation; *n* = 3) are expressed in μmol of Trolox equivalents (TE) per g of freeze-dried sample (FDS); HOCl scavenging capacity (mean ± standard deviation; *n* = 3) is expressed as IC_50_ (μg FDS/mL extract). Within each sample, different (a, b and c) letters denote significant differences between treatments at *p* < 0.05.

The FRAP values (Figure 4A) for blade samples increased on hydrolysis, although no significant differences (*p* > 0.05) were observed between the H-Prolyve and H-ProFla hydrolysates (29.59 ± 1.21 and 30.16 ± 0.99 μmol TE/g FDS, respectively). The PI samples presented considerably higher FRAP values (81.91 ± 7.76, 78.16 ± 4.97 and 73.84 ± 6.16 μmol TE/g FDS for control, H-Prolyve and H-ProFla, respectively) compared to the blade samples, although no significant differences (*p* > 0.05) were found within PI samples. These results are not in agreement with previous data reported by Stack et al. [24] for *P. dioica* PI hydrolysates (FRAP values varied between ~1 to 8 and ~4 to 29 μmol TE/g FDS, for controls and hydrolysates, respectively). In this study, the PI control and the respective hydrolysates presented considerably higher FRAP values (up to ~3 times) than those found by Stack et al. [24]. This may be explained by the different enzyme preparations used in the previous study which was a combination of Alcalase® and Flavourzyme® or differences in the starting protein profiles.

Alcalase® and Prolyve® 1000 are both *Bacillus licheniformis* proteinase preparations. Both have subtilisin activity as the main proteolytic component. Subtilisin activity is relatively nonspecific but it mediates higher specificity for aromatic and hydrophobic residues. Furthermore, Alcalase® contains a glutamyl endopeptidase activity that is not present in Prolyve® 1000. As a result, Alcalase® has been shown to be highly specific for carboxy side of glutamic acid and, at a lesser extent, to carboxyl side of aspartic acid residues [35,36]. Based on this, it appears that the combination of enzymes used had a major influence on the peptides generated and, consequently, on their FRAP activity. As mentioned, the subtilisin activity of Prolyve® 1000 may have exposed more aromatic and hydrophobic residues, like phenylalanine, tyrosine, leucine, or glutamine, which have been described as having higher antioxidant activity [37]. A study performed by Zhao et al. [38] in rice dreg protein showed that the unhydrolyzed sample presented higher reducing power compared to the hydrolysates, which is in accordance to the findings of this study.

The DPPH· inhibition ability is depicted in Figure 4B. Statistical differences (*p* < 0.05) were found in the blade sample between the control and hydrolysates (14.2 ± 0.8, 16.5 ± 0.6, and 18.1 ± 1.4 μmol TE/g FDS for control, H-Prolyve and, H-ProFla, respectively). For the PI, H-Prolyve presented higher (*p* < 0.05) DPPH· inhibition ability (48.4 ± 6.0 μmol TE/g FDS) than H-ProFla (39.5 ± 5.3 μmol TE/g FDS), but no statistical differences were observed between the control (41.2 ± 5.5 μmol TE/g FDS) and the hydrolysates. The PI samples showed more than double DPPH· inhibition capacity than the blade samples. This could be related to the higher levels of protein in the PI samples (Table 1), what is consistent with previously reported data [39]. This could potentially have resulted in the generation of a larger number of peptides and/or other compounds with DPPH· inhibition capacity that act as electron donors, converting free radicals into more stable products [39,40]. Similar results have been reported for the DPPH· inhibition effect of rice dreg protein hydrolysates. In that study, the hydrolysates presented lower radical scavenging activity than the unhydrolyzed rice dreg protein [38]. The authors suggested that the differences found in the DPPH· inhibition responses of the samples might be attributed to the amino acids’ composition and the sequence of the peptides, as well as the quantity of generated peptides, which is governed by the specificity of the enzymes used.

The ABTS·+ scavenging activity of the samples is shown in Figure 4C. The enzymatic treatment promoted a significant increase (*p* < 0.05) in the radical scavenging capacity in both blade and PI hydrolysates compared with the respective controls (up to 2.4 times). The PI samples also presented a higher ABTS·+ scavenging capacity compared to the blade samples, that may also be related to the higher protein concentration in these samples (Table 1), as previously mentioned for the DPPH· assay results [40]. Moreover, for the PI, the incubation with Flavourzyme® following Prolyve® 1000 had no effect *(p* > 0.05) on the scavenging capacity in comparison with the hydrolysate generated with Prolyve® 1000 (519.3 ± 13.53 vs. 513.5 ± 7.64 μmol TE/g FDS, respectively). This may suggest that, in this case, the enzymatic treatment might have generated peptides with similar ability to scavenge the ABTS radical. In contrast, the sequential enzymatic treatment of the blade samples, promoted a significant increase (up to 2.8 fold) in the ABTS·+ scavenging capacity of the hydrolysates compared to the control (292.9 ± 10.6 vs. 343.3 ± 11.53 μmol TE/g FDS for H-Prolyve and H-ProFla, respectively). As shown in Table 2 the controls and hydrolysate samples contained phenolic compounds which may have contributed to the observed antioxidant activity therein.

As shown in Figure 4D, blade hydrolysates presented significantly higher (*p* < 0.05) ORAC values (3054.0 ± 333.2 and 2741 ± 239.1 μmol TE/g FDS for H-ProFla and H-Prolyve, respectively) compared to the corresponding PI samples (640.10 ± 23.00 and 673.9 ± 71.541 μmol TE/g FDS for H-ProFla and H-Prolyve, respectively). These results suggested the presence of other antioxidant compounds besides proteins in the blades, which were released during hydrolysis, e.g., phenolics which are able to scavenge ROO·. These results are in accordance with those described for *P. palmata*, by Wang et al. [5] in which polyphenols were reported to contribute greatly to the peroxyl radical scavenging properties of hydrolysates. The proteolytic treatment, although depending on the enzymes used, significantly increased total phenolic content (TPC) compared to water extracts. Moreover, a higher content of phenolic compound release was observed during the conversion of proteins into small peptides and free amino acids [5]. As previously mentioned, ORAC is an in vitro antioxidant assay with biological relevance when compared with FRAP, DPPH·, and ABTS·+ scavenging assays. One of the major findings in this study was that the sequential proteolytic treatment assessed promoted the maximum antioxidant activity in the blades-derived hydrolysate H-ProFla. The synergistic antioxidant effect of polyphenols and peptides/amino acids generated might explain the results, as the ORAC values for the PI hydrolysates were similar to those observed in the ABTS·+ scavenging activity.

As can be seen in Table 2, the incubation of untreated blade sample at 4 °C for 16 h resulted in a TPC value of 2.55 ± 0.47 mg GAE/g FDS. This assay was performed in order to detect any potential endogenous proteolytic activity in blade sample that might influence further controlled enzymatic treatments. Interestingly, the incubation of the blade sample at 50 °C for 4 hours led to no further increase in TPC compared to the blade sample incubated at 4 °C for 16 h (2.55 ± 0.47 vs. 2.25 ± 0.10 mg GAE/g FDS). The hydrolysis promoted an increase in TPC (3.19 ± 0.11 to 3.42 ± 0.08 mg GAE/g FDS for H-Prolyve and H-ProFla, respectively). TPC significantly increased (*p* < 0.05) in the PI hydrolysates compared to the corresponding control (from 1.89 ± 0.11 to 4.34 ± 0.12 mg GAE/g FDS, for control and H-ProFla, respectively). On the other hand, the extent of TPC increment was less pronounce in the blade samples following hydrolysis. Based on the overall ORAC profile, blade samples were expected to present higher amounts of TPC compared to PI. In fact, enzymatic hydrolysis of *Porphyra tenera* has been previously shown to promote the release of phenolic compounds to the medium. In that case, the amount of phenolics released varied depending on the enzyme used [21]. However, it has been reported that the Folin-Ciocalteu reagent is highly reactive with a wide range of compounds, including nitrogenous compounds, namely reducing amino acids [41] and phycobiliproteins [16]. Therefore, the real TPC may be overestimated especially in the PI sample (control and hydrolysates). In addition, the alkaline extraction and isoelectric precipitation, considered as a semi-purify protein approach, may also co-extract other compounds such as phenolics. The higher TPC values in the PI H-ProFla compared to the blade H-ProFla (4.34 ± 0.12 vs. 3.42 ± 0.08 mg GAE/g FDS, respectively) may correspond to the higher protein content in the PI starting sample (Table 1).

In a previous study, the antioxidant activity of enzyme-assisted extracts obtained from an edible seaweed *Enteromorpha prolifera* using different proteases, including Flavourzyme®, was attributed, in part, to the presence of polyphenols [42]. Thus, antioxidant activity can be the result of synergistic effects of the constituents in the hydrolysate.

As shown in Figure 4E, all samples, both the blade and PI controls and their corresponding hydrolysates, presented scavenging activity against HOCl, reported as calculated IC_50_ values in µg/mL. All the test samples had the ability to prevent HOCl-induced oxidation of DHR-123 in a concentration-dependent manner (Figure S1 in Supplementary material). Furthermore, all test samples had considerably lower IC_50_ values (<32.65 µg FDS/mL) compared to Trolox (116.5 µg /mL), indicating higher scavenging activity. Compared to the control, the blade hydrolysates were more effective HOCl scavengers (11.02 ± 1.83, 12.81 ± 2.2 and 15.51 ± 1.79 µg FDS/mL for H-ProFla, H-Prolyve and control, respectively). Likewise, the PI derived H-Prolyve and H-ProFla also presented significantly lower IC_50_ values (13.86 ± 2.42 and 20.82 ± 3.1 µg FDS/mL, respectively) compared to the control (32.65 ± 1.94 µg FDS/mL). The results indicated that the greater the extent of hydrolysis, the lower the IC_50_ obtained. To the best of our knowledge, this is the first time that *P. dioica*-derived hydrolysates have been reported to possess potent HOCl scavenging activity.

Enzyme-assisted hydrolysis has been utilized previously to prepare water-soluble extracts from seaweed with antioxidant activity. Ahn et al. [43] employed a combination of carbohydrases and proteases with the brown species *Scytosiphon lomentaria* and found that the enzymatic extracts exhibited strong scavenging activity on hydroxyl, alkyl and DPPH·, on a concentration-dependent manner. *Porphyra columbina* hydrolyzed using different enzymes/enzymatic combinations (trypsin alone, Alcalase® alone, trypsin followed by Alcalase® and Alcalase® followed by trypsin) have previously been shown to exhibit antioxidant activity [16]. All *Porphyra columbina* hydrolysates were shown to exhibit higher antioxidant capacity in DPPH·, ORAC and ABTS·+ assays than that observed with the undigested substrate (previously extracted protein fraction with molecular mass > 10 kDa). Furthermore, *P. dioica*-derived hydrolysates generated with a combination of Alcalase® 2.4 L and Flavourzyme® 500L were also shown to mediate potent antioxidant activity [24]. However, this is the first study reporting the antioxidant properties of *P.dioica* blades and PI derived hydrolysates using the combination of enzymes Prolyve® 1000 and Flavourzyme®.

## 3. Materials and Methods

### 3.1. Reagents and Standards

Flavourzyme® (protease from *Aspergillus oryzae*) was from Sigma-Merck (Dublin, Ireland) and Prolyve® 1000 (protease from *Bacillus licheniformis*) was kindly provided by Lyven Enzyme Industrielles (Caen, France). Trinitrobenzenesulphonic acid (TNBS) was from Fisher Scientific (Dublin, Ireland). HPLC-grade acetonitrile and water were from VWR International (Dublin, Ireland). Leucine, gallic acid, Trolox, 2,2’-azobis-2-methyl-propanimidamide (AAPH), fluorescein, 2,2-diphenyl-1-picrylhydrazyl (DPPH), L-leucine, 2,2′-azinobis-(3-ethylbenzothiazoline-6-sulfonate) (ABTS·+), Folin-Ciocalteu reagent, dihydrorhodamine 123 (DHR), sodium hypochlorite solution (with 4% available chlorine), and all other reagents were supplied by Sigma-Merck.

### 3.2. Samples

#### 3.2.1. Algal Biomass

Organic certified biomass produced in an integrated multitrophic aquaculture (IMTA) system was provided by ALGAplus Ltd. (Ria de Aveiro, Portugal, 40°36’43” N, 8°40’43” W). The algal biomass was collected in December 2018. Dried samples were ground using a Thermomix® mill (Vorwerk Thermomix TM5, Asbach, Germany), stored in vacuum-sealed bags and these were stored in the dark until further use.

#### 3.2.2. Crude Protein Extracts

The protein was extracted from the biomass according to a previously described methodology that combines aqueous and alkaline protein solubilization [28]. The protein isolate was then generated following isoelectric precipitation at pH 4.5 and neutralization [24]. Samples were freeze-dried and stored at room temperature (20 ± 0.2 °C) in darkness in an air-tight container until further use.

#### 3.2.3. Determination of Nitrogenous Composition

TN, NPN, and PN content of blades and PI were determined by a modified macro-Kjeldahl procedure as previously described by Connolly et al. [44]. The protein content was then estimated based on the N × 5.00 converting factor [29].

#### 3.2.4. Hydrolysate Generation

A two-step hydrolysis protocol using different proteolytic preparations (Prolyve 1000® and Flavourzyme®) was used to generate the hydrolysates from the milled biomass (blades) and the PI. Biomass was hydrated (1:20, *w*/*v*) at 4 °C, overnight, with gentle stirring. Likewise, an aqueous dispersion of PI (1:20, *w*/*v*) was stirred for 1 h, at room temperature. The mixtures were then equilibrated at 50 °C, the pH adjusted to 8.0 with 1.0 M NaOH prior incubation with Prolyve 1000® at an enzyme-to-substrate ratio (E:S) 1% (*v*/*w* of protein) for 120 min, ultimately leading to a hydrolysate termed H-Prolyve. Subsequently, H-Prolyve was incubated with Flavourzyme® under the same conditions (pH 8, 50 °C, 1% (*v*/*w*, protein) E:S, 120 min). This led to a hydrolysate termed as H-ProFla. The pH of the reaction mixture was maintained constant (pH 8) throughout hydrolysis using a pH-STAT (Titrando 842, Tiamo 1.4 Metrohm, Dublin, Ireland). Enzymes were then inactivated at 80 °C for 20 min. The supernatants obtained after centrifugation (11,950× *g*, 20 min, 10 °C) of both hydrolysates and controls (samples treated under the same conditions but without added proteases) were freeze-dried and stored at room temperature in darkness until further analysis.

#### 3.2.5. Characterization of the Hydrolysates

The extent of hydrolysis was assessed using TNBS as described by Le Maux et al. [45]. The amount of free amino groups released during the hydrolysis was determined using a leucine standard curve (0–2.0 mM, R^2^ = 0.9972). Analysis were performed in triplicate and results presented as mg N/g freeze-dried sample (FDS). The molecular mass distribution of the samples was performed by gel permeation high performance liquid chromatography (GP-HPLC) according to Spellman et al. [30] and peptide profiles were determined by reverse-phase ultra-performance liquid chromatography (RP-UPLC) as described by Cermeño et al. [19]. Total phenolic compounds (TPC) were estimated according to the procedure described by Nunes et al. [46].

#### 3.2.6. Preparation of Samples and Antioxidant Bioassays

Intact biomass, controls, and freeze-dried hydrolysates (from both blades and PI) were dispersed at 20 mg/mL in deionized water or in the appropriate buffer for each bioassay and the samples were centrifuged (21,250× *g*, 5 min, at room temperature; Hettich Universal 320R, Andreas Hettich GmbH & Co. KG, Germany) to remove insoluble material. Supernatants were then diluted as needed. Hydrolysates and controls were characterized for their in vitro antioxidant activity using a range of bioassays. The oxygen radical absorbance capacity (ORAC) and ferric reducing activity power (FRAP) were assessed as described by Harnedy and FitzGerald [25]. Samples were also assessed for their ability to scavenge DPPH· [47], HOCl [1] as well as for their Trolox equivalent antioxidant capacity (TEAC) using potassium persulfate to generate the radical (ABTS·+), according to the procedure described by Kleekayai et al. [48]. Experiments were performed in triplicate (*n* = 3).

#### 3.2.7. Statistical Analysis

The results are presented as the mean ± standard deviation (SD) of at least three independent experiments. Data was analyzed by one-way analysis of variance (ANOVA) at a significance level of *p* < 0.05, followed by multiple comparisons using Tukey’s post-hoc test. GraphPad Prism 5 software (GraphPad, San Diego, CA, USA) was used for the statistical analysis.

## 4. Conclusions

This study focused on characterizing the ability of *P. dioica*-derived ingredients to display antioxidant activity. In general, protein hydrolysates generated from *P. dioica* biomass/blades presented higher antioxidant activity when compared to unhydrolyzed controls. A similar trend was observed for protein isolate (except for the FRAP assay where the reducing power slightly decreased following hydrolysis).

The results showed that the hydrolysis using this specific combination of enzymes (Prolyve® 1000 plus Flavourzyme®) can be used to produce functional hydrolysates/peptides from *P. dioica* with potent antioxidant activity when evaluated by different antioxidant assays (and thus different antioxidant mechanisms). As previously reported, there are advantages in using enzyme-assisted hydrolysis to obtain natural antioxidant compounds from seaweeds—besides being a scalable industrial production process, it allows the generation of water-soluble antioxidant ingredients, namely peptides.

In sum, the generation of *P. dioica* derived hydrolysates with enhanced antioxidant properties can be of potential value in the generation of functional food ingredients/nutraceuticals. However, fractionation, identification and further characterization of peptides from the most potent hydrolysates is still required in order to elucidate the role of specific *P. dioica* peptides as antioxidant agents.

## Figures and Tables

**Table 1:** Total nitrogen, non-protein nitrogen, protein nitrogen, and protein content of blades and protein isolate (PI) from *Porphyra dioica.*

Sample	NPN	PN	TN	Protein*
	(% dw)	(% dw)	(% dw)	(% dw)
Blades	0.99 ± 0.07 ^a^	3.69 ± 0.20 ^b^	4.60 ± 0.18 ^b^	23.09 ± 0.90 ^b^
PI	0.06 ± 0.001 ^b^	8.26 ± 0.27 ^a^	10.10 ± 0.30 ^a^	50.30 ± 1.52 ^a^

Results are presented as mean ± SD of 3 independent determinations. NPN: non-protein nitrogen; PN: protein nitrogen; TN: total nitrogen; dw: dry weight. Different letters (a and b) in the same column denote significant differences at *p* < 0.05. * Protein content was estimated using a Kjeldahl nitrogen to protein conversion factor of 5.00 [29].

**Table 2:** Total phenolic content (TPC) of blades and protein isolate from *Porphyra dioica* following 2 h hydrolysis at 50 °C with Prolyve® 1000 (H-Prolyve) and 4 h with Prolyve® 1000 plus Flavourzyme® (H-ProFla).

Treatment	Blades (mg GAE/g FDS)	Protein isolate (mg GAE/g FDS)
No treatment (4 °C, 16 h)	2.55 ± 0.47 ^a^	na
Control	2.25 ± 0.10 ^a^	1.89 ± 0.11 ^a^
H-Prolyve	3.19 ± 0.11 ^b^	3.96 ± 0.09 ^b^
H-ProFla	3.42 ± 0.08 ^b^	4.34 ± 0.12 ^b^

Results (mean ± standard deviation, *n* = 3) are presented as mg gallic acid equivalents (GAE) per g of freeze-dried sample (mg GAE/g FDS). Different superscript letters (a and b) within the same column represent significant differences between treatments for each sample (blades or PI), individually, at *p* < 0.05. na: not applicable.

## Supplementary Materials

The following are available online at https://www.mdpi.com/1420-3049/25/12/2838/s1, Figure S1. Concentration-response plots for the scavenging capacity of: *P. dioica* blades (A) and protein isolate (B) with their corresponding Prolyve® 1000 (H-Prolyve), and Prolyve® 1000 plus Flavourzyme® (H-ProFla) hydrolysates; C, Trolox. Error bars represent standard error from at least three independent experiments, assayed at six different concentrations. The control sample corresponds to blades and protein isolate incubated at 50 °C for 4 h without enzymes.
