# Supplementary material for: Enzymatic Modification of Porphyra dioica-Derived Proteins to Improve their Antioxidant Potential
Source: Molecules. 2020 Jun 19;25(12):2838. doi: 10.3390/molecules25122838 (PMC7355851; doi:10.3390/molecules25122838)
Supplement: Supplementary file 1 [file molecules-25-02838-s001.pdf]

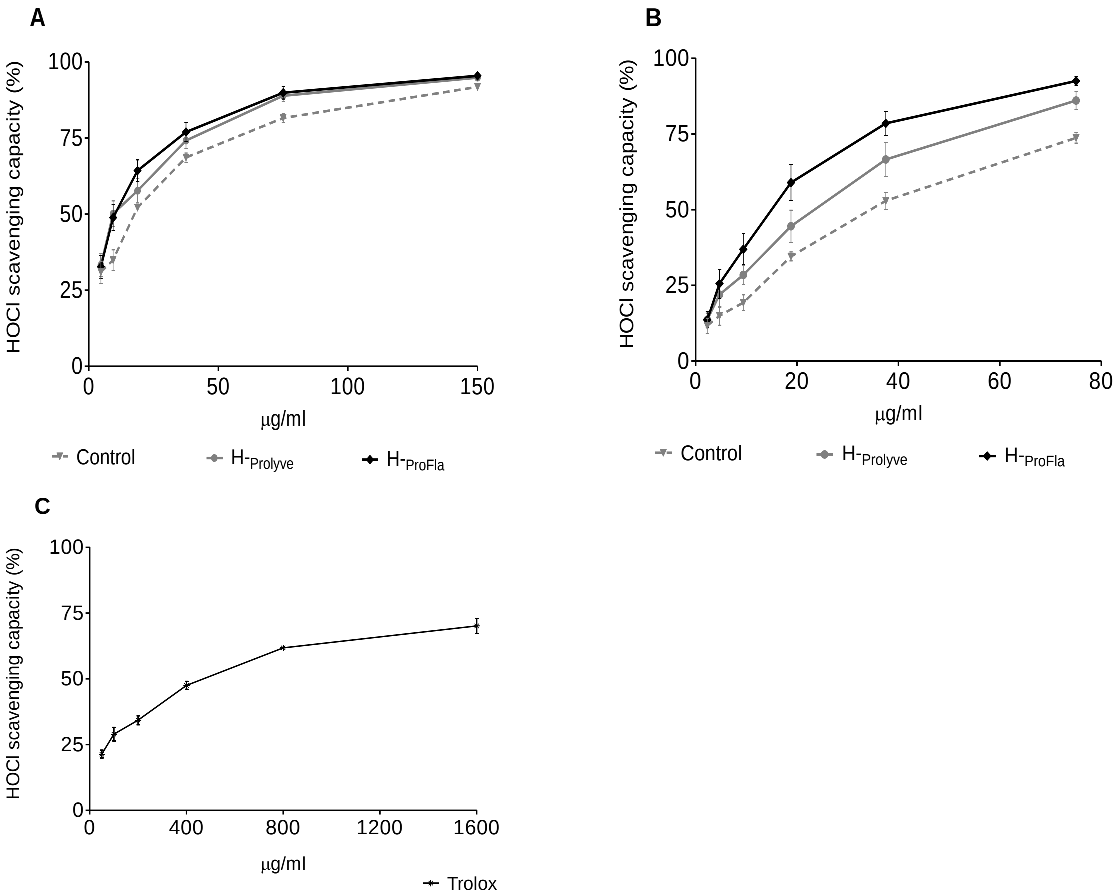


**Figure S1.** Concentration-response plots for the scavenging capacity of: *P. dioica* blades (**A**) and protein isolate; (**B**) with their corresponding Prolyve® 1000 (H-Prolyve), and Prolyve® 1000 plus Flavourzyme® (H-ProFla) hydolysates; (**C**) trolox. Error bars represent standard error from at least three independent experiments, assayed at six different concentrations. The control sample corresponds to blades and protein isolate incubated at 50 °C for 4 h without enzymes.
